# Influence of air temperature and implemented veterinary measures on the incidence of human salmonellosis in the Czech Republic during 1998–2017

**DOI:** 10.1186/s12889-020-10122-8

**Published:** 2021-01-06

**Authors:** Jan Kynčl, Michaela Špačková, Alena Fialová, Jan Kyselý, Marek Malý

**Affiliations:** 1 Department of Infectious Diseases Epidemiology, Centre for Epidemiology and Microbiology, National Institute of Public Health, Šrobárova 49/48, 100 00 Prague, Czech Republic; 2 Department of Epidemiology and Biostatistics, Third Faculty of Medicine, Charles University, Prague, Czech Republic; 3 Department of Biostatistics, National Institute of Public Health, Prague, Czech Republic; 4 Institute of Atmospheric Physics, Czech Academy of Sciences, Prague, Czech Republic; 5 Faculty of Environmental Sciences, Czech University of Life Sciences, Prague, Czech Republic

**Keywords:** Salmonellosis, Temperature, Weather, Veterinary measures, Gastrointestinal infections

## Abstract

**Background:**

The aim of our study was to analyse the influence of air temperature and implemented veterinary measures on salmonellosis incidence in the Czech Republic (CZ).

**Methods:**

We conducted a descriptive analysis of salmonellosis as reported to the Czech national surveillance system during 1998–2017 and evaluated the influence of applied veterinary measures (started in January 2008) on salmonellosis incidence by comparing two 9-year periods (1998–2006, 2009–2017). Using a generalized additive model, we analysed association between monthly mean air temperature and log-transformed salmonellosis incidence over the entire twenty-year period.

**Results:**

A total of 410,533 salmonellosis cases were reported during the study period in the CZ. Annual mean incidences of salmonellosis were 313.0/100,000 inhabitants before and 99.0/100,000 inhabitants after implementation of the veterinary measures. The time course of incidence was non-linear, with a sharp decline during 2006–2010. Significant association was found between disease incidence and air temperature. On average, the data indicated that within a common temperature range every 1 °C rise in air temperature contributed to a significant 6.2% increase in salmonellosis cases.

**Conclusions:**

Significant non-linear effects of annual trend, within-year seasonality, and air temperature on the incidence of salmonellosis during 1998–2017 were found. Our study also demonstrates significant direct effect of preventive veterinary measures taken in poultry in reducing incidence of human salmonellosis in the CZ. The annual mean number of salmonellosis cases in the period after introducing the veterinary measures was only 32.5% of what it had been in the previous period.

## Background

*Salmonellae* are among the most common bacterial foodborne pathogens worldwide and the second most commonly reported zoonotic agent in the European Union (EU), with notification rates of 20.1 cases per 100,000 population in 2018 [1]. *Salmonellae* caused 31% of all food- and water-borne outbreaks in the EU in 2018 [1]. In the Czech Republic (CZ), *Salmonella* was the leading causative agent of acute gastroenteritis until 2006. Since 2007, it has been the second most common after campylobacteriosis [2].

*Salmonella* spp. are Gram-negative bacteria belonging to the *Enterobacteriaceae* family. Subspecies of species *S. enterica* are subdivided into more than 2500 serovars, many of which commonly colonize animals and infect humans [3, 4]. Different *Salmonella* serovars are also categorised as typhoidal (*S.* Typhi and *S.* Paratyphi) and non-typhoidal (e.g. *S.* Enteritidis, *S.* Typhimurium, and *S.* Infantis), based on causative agent and clinical symptoms [3]. In our study, we only consider non-typhoidal human salmonellosis as this is the most burdensome class of *Salmonella* infections in humans within developed countries.

*Salmonella* usually causes gastroenteritis in humans. Systemic infections are rare [3]. Incubation periods of *Salmonella* range from 6 to 72 h (most usually 12–36 h) [5]. *Salmonella* bacteria tolerate various environmental conditions. They can grow at temperatures ranging from 8 to 45 °C and in pH range 4.0–9.0 [6]. The bacteria can persist for a long time in feed mill environments [7].

It is estimated that 86% of human *Salmonella* infections are of foodborne origin [8]. Mass production and distribution of food disseminates pathogens rapidly [8]. The foods most commonly associated with strong-evidence salmonellosis outbreaks in Europe are eggs, bakery products, mixed food, pig meat and products thereof, and poultry meat, followed by other food vehicles including vegetables and fruits [1]. In food, the highest levels of *Salmonella*-positive samples have occurred in poultry meat and other minced meat intended to be cooked before consumption [1]. Cross-contamination may of course also occur. The most important source of human salmonellosis at the EU level during 2007–2009 was estimated to be the laying hen reservoir (i.e. eggs), followed by pigs [9].

Several possibilities exist for controlling *Salmonella* in animals. Effort to reduce *Salmonella* in poultry is coordinated through EU control programmes. The basic law on the control of *Salmonella* and other specific foodborne zoonotic agents is Regulation (EC) No 2160/2003. Monitoring of *Salmonella* spp. along the food chain is conducted in accordance with Regulation (EC) No 2073/2005, which lays down food safety and process hygiene criteria [1]. Implementation of the general and specific hygiene measures is based on Regulation (EC) No 852/2002 [1]. In the CZ, the new veterinary strategies for reduction and control of *Salmonella* in poultry in accordance with EU legislation were officially implemented in January 2008 (although some of these had been started 1 year earlier). These new programmes for *Salmonella* reduction are conducted by the Czech State Veterinary Administration, a public administrative body under the Ministry of Agriculture, and are mainly based on principles of good husbandry, implementing consistent sanitation and zoohygienic measures, and ensuring the provision of healthy feed and water. The programmes are aimed at reducing the prevalence of *Salmonella* in poultry and the environment and its transmission to humans via contaminated food. Four categories of poultry are monitored, including breeding stock imported from abroad and animals being produced for transport abroad. Details of the national programmes are available from the State Veterinary Administration [10]. Mandatory vaccination is another tool for controlling the occurrence of *Salmonella* in poultry.

Pork is also an important source causing human salmonellosis. Eradicating *Salmonella* from pig farms can be both difficult and costly [11]. Regulation (EC) No. 218/2014 requires that competent authorities evaluate the implementation of slaughterhouse operators’ own checks for presence of *Salmonella* in pig carcasses at slaughterhouses [1]. Other than as described above, data of food, animals, and feedstuffs are not collected in a harmonised manner, although these do still need to be monitored in accordance with Directive 2003/99/EC on the monitoring of zoonoses [1]. Preventive actions, including testing for *Salmonella*, are mainly directed to avoiding contamination at feed mills and on farms. Along with technical and hygienic measures, vaccination is another possibility to prevent *Salmonella* colonization in swine herds [12].

Higher air temperatures may lead to increased foodborne illness. Within the range 7.5–37 °C, *Salmonella* spp. multiply in food in direct proportion to changing temperature [13]. In the absence of any control measures, therefore, increased air temperatures may accelerate bacterial reproduction at various points along the food chain, thus making the consequences of subsequent ingestion more severe. Air temperature may also influence people’s behaviour in ways that affect the chances for foodborne illness to occur (such as to prompt buying of ready-to-eat food or barbecuing in warmer weather) [13]. Increased outdoor recreational activity may also increase the likelihood that people will be exposed to environmental sources of *Salmonella* [13]. Many studies have demonstrated positive associations between temperature and foodborne illness in a variety of geographical settings [13–16]. Linear associations have been noted between temperature and notifications of salmonellosis in European countries and Australia [17].

The aim of our study was to analyse the influence of air temperature and implemented veterinary measures on salmonellosis incidence in the CZ.

## Methods

Non-typhoidal salmonellosis is a mandatorily notifiable disease in the CZ and the reporting covers the whole population. The regional public health authorities therefore notify case-based data and related metadata of salmonellosis into the national surveillance system for infectious diseases (EpiDat) that is administered by the National Institute of Public Health. EpiDat contains all notified probable and confirmed cases that meet the case definition in accordance with Commission Implementing Decision (EU) No 2119/98/EC (of 19 March 2002). Specifically, any person meeting the clinical criteria with an epidemiological link is reported as a probable case and any person meeting the clinical and laboratory criteria is reported as a confirmed case. We analysed data on sporadic cases and household outbreaks of human non-typhoidal salmonellosis. Epidemics in the CZ are determined by the local health authorities conducting epidemiological investigation in the field while considering number of cases, mode of transmission, and common vehicle or source. Cases designated as epidemic were excluded due to their different characteristics and pattern of spreading. We analysed monthly case counts (by date of onset) notified during the period 1998–2017. Monthly mean air temperatures across the territory of CZ were provided by the Czech Hydrometeorological Institute [18]. Data on population were obtained from the Czech Statistical Office [19]. For descriptive purposes, data from two nine-year periods (1998–2006, 2009–2017) were compared while years 2007–2008, when the measures started and were implemented, were excluded from the comparison.

The association between disease incidence and a set of explanatory variables (year, month, and temperature) was studied using a generalized additive model (GAM) [20] on data from the entire twenty-year period. Because the incidence is characterized by counts overdispersed relative to the Poisson distribution, a semiparametric negative-binomial GAM was used with smooth and potentially non-linear effects of annual trend, within-year seasonality, and monthly mean air temperature. The model fitting was based on the penalized likelihood and performed in R, version 3.4.3 [21] using the *mgcv* package. The final model was selected with the aid of the Akaike information criterion. All tests were evaluated at the 5% significance level.

The final model is specified as follows:

$$ {N}_t\sim \mathrm{NB}\left({\mu}_t,\theta \right) $$

and

$$ \log\ {\mu}_t={\beta}_0+{s}_{Year}\left({y}_t\right)+{s}_{Season}\left({m}_t\right)+{s}_{Temp}\left({T}_t\right), $$

where:

*t* is index of the month,

*N*_*t*_ is the number of salmonellosis cases in month *t*,

*μ*_*t*_ is the negative binomial intensity of salmonellosis occurrence,

*θ* is a parameter for overdispersion, where $\mathrm{Var}\ \left({\upmu}_t\right)={\upmu}_t+{\upmu}_t^2/\uptheta$,

*β*_0_ is the intercept term corresponding to the baseline level,

*s*_*Year*_ is smooth marginal effect of the trend over years estimated as thin-plate spline,

*s*_*Season*_ is smooth effect of seasonality (in monthly resolution) estimated as cyclic cubic regression spline, and

*s*_*Temp*_ is smooth marginal effect of temperature estimated as thin-plate spline.

Smooth components are presented along with 95% pointwise confidence intervals.

## Results

In total, 440,470 salmonellosis cases were reported in the CZ during the study period. After excluding 29,937 (6.8%) epidemic cases, the remaining 410,533 sporadic and family outbreak salmonellosis cases were analysed. Of these cases, 228,249 (55.6%) occurred among children and adolescents up to 19 years of age. The sample comprised 196,780 (47.9%) males and 213,753 (52.1%) females.

For a basic assessment of the conditions before and after the implementation of veterinary measures, the following analysis compared two nine-year periods, excluding the transitional period 2007–2008. In the first studied time period (1998–2006), we analysed 288,746 cases: 139,034 males (48.2%) and 149,712 females (51.8%), mean age 23.4 years. In the second period (2009–2017), we analysed 93,826 cases: 44,596 males (47.5%) and 49,230 females (52.5%), mean age 24.4 years. This means that the annual average number of salmonellosis cases occurring after introduction of the veterinary measures was only 32.5% of that observed in the previous period. The two studied periods did not differ in the proportions of cases in individual age groups. The mean annual incidence of salmonellosis was 313.0/100,000 inhabitants before and 99.0/100,000 inhabitants after implementation of the veterinary measures.

**Fig. 1:**
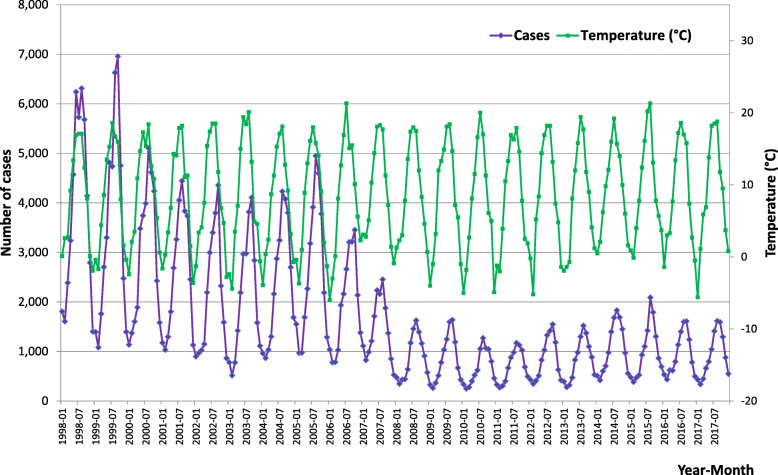
Absolute numbers of salmonellosis cases and mean air temperature, by month, Czech Republic, 1998–2017

**Fig. 2:**
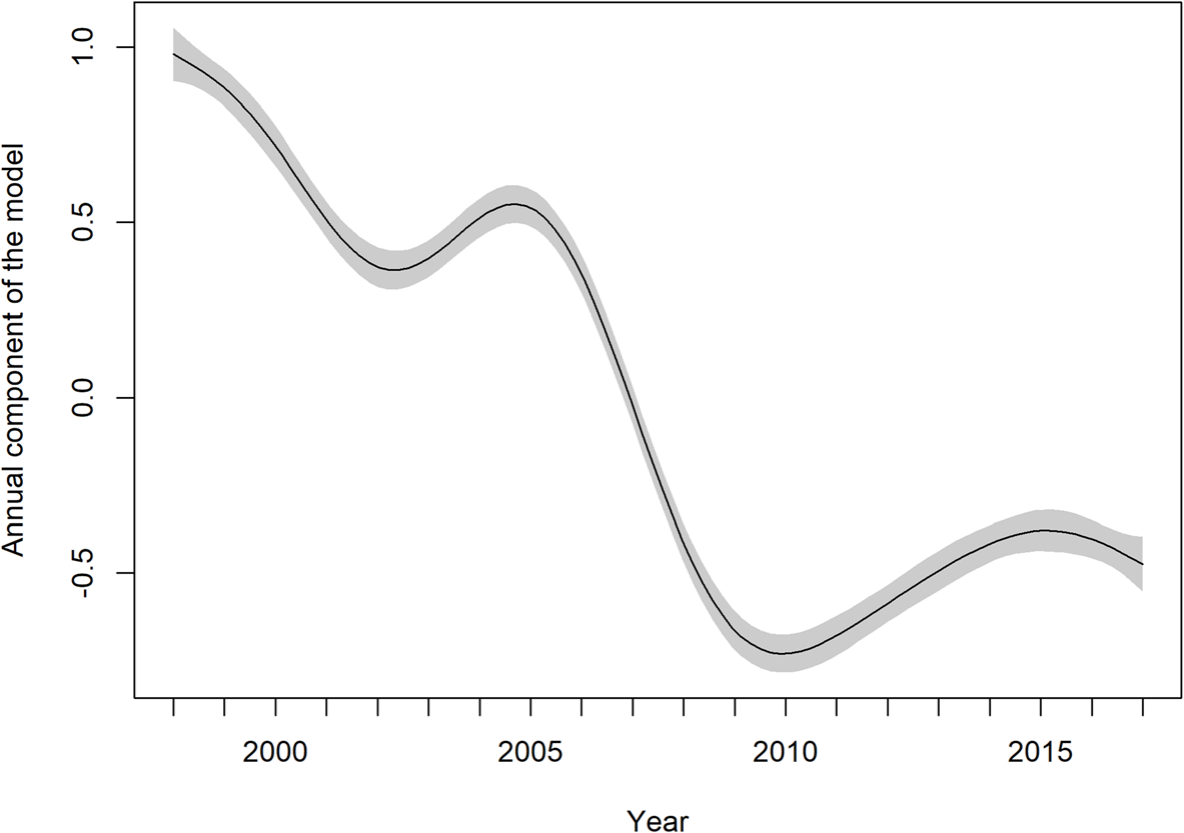
Annual component of the model with 95% pointwise confidence interval

**Fig. 3:**
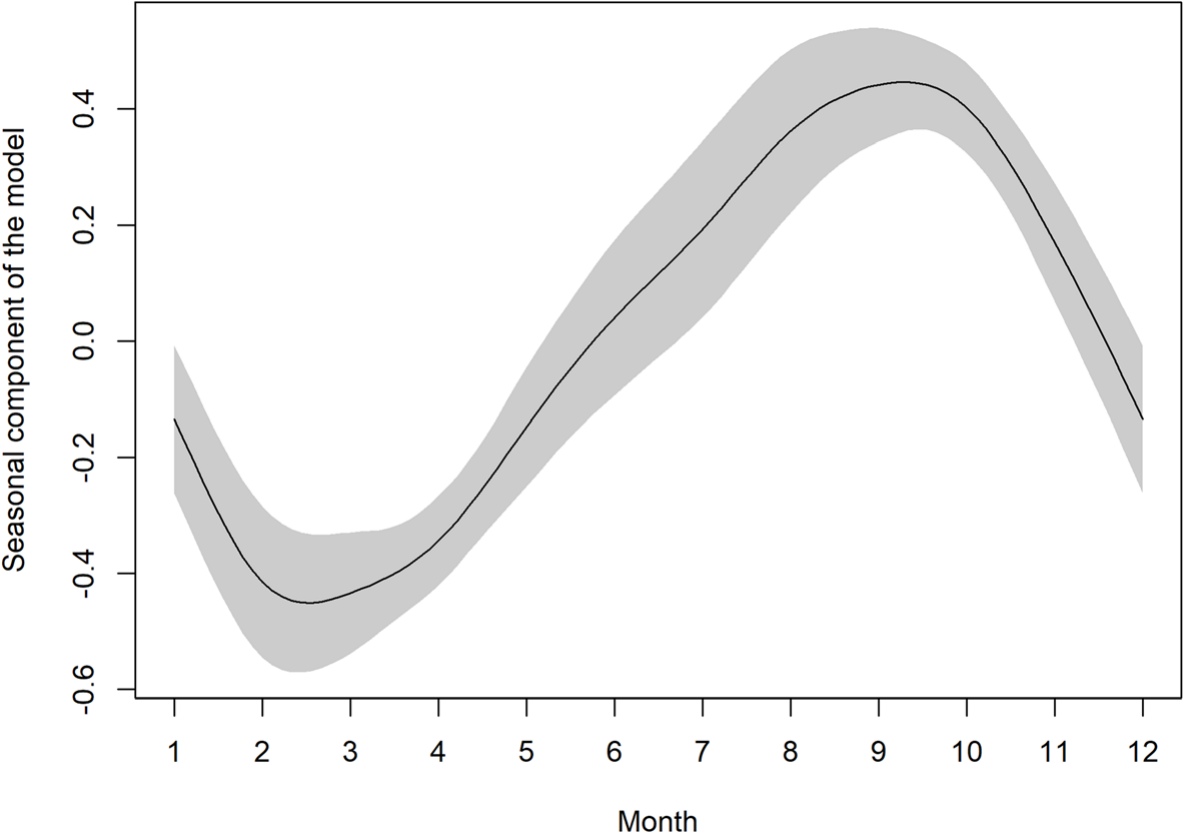
Seasonal component of the model with 95% pointwise confidence interval

**Fig. 4:**
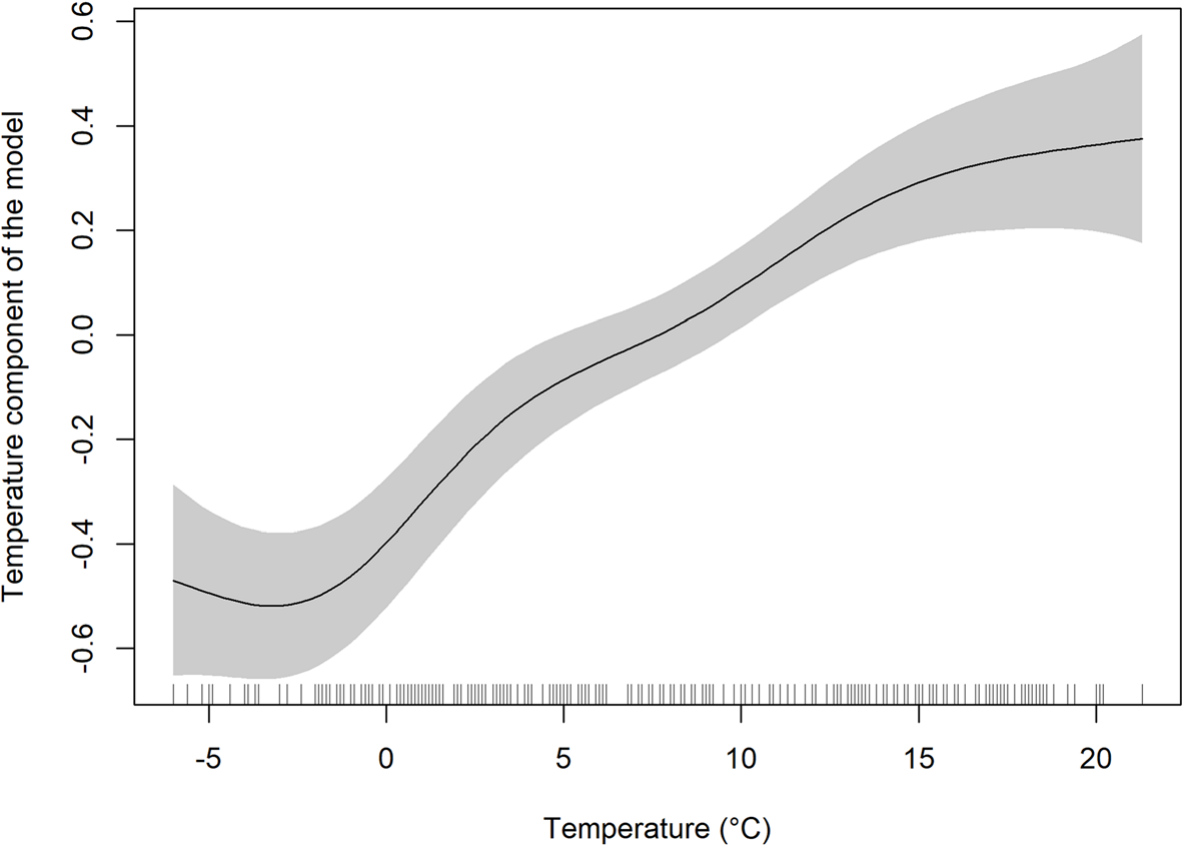
Temperature component of the model with 95% pointwise confidence interval and marks on the x-axis indicating the distribution of the data

Overall trends were analysed in the entire data set. As clearly visible in Fig. 1, there was a substantial decrease in the number of cases during 2007–2008. All predictors included in the GAM model are statistically significant (*p* < 0.001). The adjusted R^2^ (coefficient of determination) of 96.2% that was calculated indicates that the model explains a substantial part of the data variability. A detailed look at the output of the GAM model shows that the course of incidence over time is non-linear. After a slight decline at the beginning of the study period, the sharpest decline occurred in 2006–2009. This was followed by a slight increase and stabilization at a level that is substantially lower compared to the levels observed before imposition of the measures (Fig. 2). The seasonal component (Fig. 3) shows a clear within-year trend with a minimum in February and March and a maximum in August and September. The dependence of disease incidence on temperature is depicted by an S-shaped curve. Figure 4 shows that the relationship is almost linear in the temperature range 0–15 °C within which the vast majority of data falls, as indicated by the distribution marks on the x-axis in the form of a rug plot. At mean monthly temperature extremes, the curve approaches the asymptotes. On average, the data indicate that within the aforementioned temperature range every 1 °C increase in monthly air temperature contributed to a significant 6.2% increase in salmonellosis cases.

## Discussion

Salmonellosis has been a mandatorily notifiable disease in the CZ since 1951. Its incidence slowly increased in the country until the 1980s. Thereafter, a sharp increase in salmonellosis incidence was observed until 1995, consistent with a similar rise in other European countries [22, 23]. The most probable causes of this sharp rise were an increase in pathogen invasiveness and the appearance of new phagotypes of *Salmonella* Enteritidis in the factory-style farming within the European poultry sector [24]. After 1995, salmonellosis cases in the CZ declined slowly and with certain deviations until 2005. Thereafter, in accordance with international strategies for reducing *Salmonella* in poultry that were implemented in the CZ between 2007 and 2008, we observed a sharp decline of salmonellosis until 2010. As the decline was already apparent before the measures entered into force, it is clear that the poultry producing companies had been preparing for them in advance. Sporadic cases of salmonellosis in the CZ during 2009–2017 averaged 99.0/100,000 inhabitants per year, while the total incidence of salmonellosis (including epidemic cases) was 103.9/100,000 per year. The present-day reduced incidence of human salmonellosis cases in the CZ is notably the result of consistent *Salmonella* control in the country. Nevertheless, salmonellosis cases reported in the CZ are still five times higher than the EU average. The average EU notification rate was 20.1 cases per 100,000 population in 2018 while in the CZ it was 102.7/100,000 population [1]. Notification rates in neighbouring Slovakia (124.8/100,000 population) are even higher compared to those in the CZ, and the lowest rates are being reported by Cyprus, Greece, Italy and Portugal (≤ 6.0/100,000 population) [1]. It is not clear whether intercountry variation in reported incidence of salmonellosis is due to (a) different levels of true morbidity, (b) different methods of detection and reporting, or (c) a combination of the two.

Confirming the findings of previous studies [13, 15, 25–27], substantial positive association was found between air temperature and non-typhoidal *Salmonella* infections in the CZ during 1998–2017. Our calculations put this increase at 6.2% more salmonellosis cases per 1 °C rise within the monthly mean temperature range of 0–15 °C. This finding is underscored by the facts that, during the latter period of the analysed time span, the highest number of salmonellosis cases in summer was reported in 2015, which had the hottest summer (July–August) ever recorded, whereas the lowest number of cases in summer was reported in the coolest summer of 2011. It is known that higher temperatures enable quicker replication of *Salmonella* which is able to multiply at ambient temperatures with generation time as low as 20 min [28, 29]. Heat stress can induce enteritis in chickens with Salmonella present in their guts and its spread to other organs [28, 30]. The potential risk arises through cross-contamination by food-handling with human cases appearing after consummation of contaminated meat [30]. It is therefore likely that with rising temperatures the risk of salmonellosis occurrence in humans will be increasing and hence also will the importance of preventive measures.

Preventive veterinary and food safety measures are the most effective way to protect the public against the disease. EU-coordinated control programmes in poultry for combatting *Salmonella*’s spread through food consumption have constituted a major success story in the EU. To be most effective, efforts to reduce transmission of *Salmonellae* via food and other routes should be implemented on a comprehensive scale [8].

### Limitations of the study

We did not consider incubation period of the disease in this analysis. Inasmuch as we analysed the monthly counts of cases by date of disease onset in relation to the monthly means of air temperature, we did not consider the incubation period of non-typhoidal *Salmonella* (6–72 h, usually 12–36 h) to be a relevant factor for the analysis. This might very well be relevant in studies where weekly temperature or data for even shorter time periods (days) would be analysed.

Underreporting may generally result in underestimation of the true disease burden, and that applies also for the CZ. The Czech national reporting system has been well established already since the 1950s, and therefore we do not expect that substantial changes in underreporting rate during the study period would have biased the results. We assume that for analysing association between the disease incidence and air temperature, as well as in relation to the implemented veterinary measures, the precise number of cases is not the most relevant factor. More important are the actual trends. Nonetheless, slight demographic changes in the Czech population and food consumption patterns during the study period might be a limitation, as we did not control for these potential biases.

## Conclusions

Statistical analysis based on the GAM model showed significant non-linear effects of annual trend, within-year seasonality, and air temperature on the incidence of non-typhoidal *Salmonella* infections in the CZ during 1998–2017. The highest incidence corresponds to the highest mean temperatures above 15 °C. Our study also demonstrates significant direct effect of preventive veterinary measures taken in relation to poultry in reducing human salmonellosis in the CZ. The importance of these measures is likely to grow with increasing air temperatures. The annual average number of salmonellosis cases occurring after introduction of the veterinary measures was only about one-third of that observed in the previous period.

## Data Availability

The data for human salmonellosis that support the findings of this study are available from the national surveillance system for infectious diseases (EpiDat) that is administered by the National Institute of Public Health. The data are available also from the authors upon reasonable request and with permission of the National Institute of Public Health, namely from Jan Kynčl, M.D., Ph.D. at email address jan.kyncl@szu.cz. Monthly mean air temperatures are publicly provided by the Czech Hydrometeorological Institute on its web pages. Data on population characteristics are publicly available on the web pages of the Czech Statistical Office. Veterinary measures are described on the web pages of the Czech State Veterinary Administration.

## References

[1] European Food Safety Authority. European Centre for Disease Prevention Control. The European Union one health 2018 zoonoses report. EFSA J. 2019;17(12):e05926. 10.2903/j.efsa.2019.5926 PMC7055727 32626211

[2] Špačková M, Gašpárek M (2018). Analysis of the most common food-and water-borne diseases in the Czech Republic, 2007-2017. Praktický lékař.

[3] Wen SC, Best E, Nourse C (2017). Non-typhoidal Salmonella infections in children: review of literature and recommendations for management. J Paediatr Child Health.

[4] Hendriksen RS, Vieira AR, Karlsmose S, Lo Fo Wong DM, Jensen AB, Wegener HC (2011). Global monitoring of Salmonella serovar distribution from the World Health Organization global foodborne infections network country data Bank: results of quality assured laboratories from 2001 to 2007. Foodborne Pathog Dis.

[5] Heymann DL (2015). Control of communicable diseases manual.

[6] Hanes D, Miliotis MD, Bier JW (2003). Nontyphoid Salmonella. International handbook of food-borne pathogens.

[7] Alali WQ, Ricke SC. The ecology and control of bacterial pathogens in animal feed. In: Fink-Gremmels J, editor. Animal feed contamination. Effects on livestock and food safety. Cambridge: Woodhead Publishing Series in Food Science, Technology and Nutrition; 2012. p. 35-55.

[8] Majowicz SE, Musto J, Scallan E, Angulo FJ, Kirk M, O'Brien SJ (2010). The global burden of nontyphoidal Salmonella gastroenteritis. Clin Infect Dis.

[9] De Knegt L, Pires SM, Hald T (2015). Attributing foodborne salmonellosis in humans to animal reservoirs in the European Union using a multi-country stochastic model. Epidemiol Infect.

[10] The State Veterinary Administration of the Czech Republic (2019). The national programmes to reduce prevalence of Salmonella in poultry.

[11] Hald T (2010). EFSA Panel on Biological Hazards; Scientific Opinion on a Quantitative Microbiological Risk Assessment of Salmonella in slaughter and breeder pigs.

[12] Wales A, Davies R (2017). Salmonella vaccination in pigs: a review. Zoonoses Public Health.

[13] Lake I, Gillespie I, Bentham G, Nichols G, Lane C, Adak G (2009). A re-evaluation of the impact of temperature and climate change on foodborne illness. Epidemiol Infect.

[14] Bentham G, Langford IH (2001). Environmental temperatures and the incidence of food poisoning in England and Wales. Int J Biometeorol.

[15] Kovats R, Edwards S, Hajat S, Armstrong B, Ebi K, Menne B (2004). The effect of temperature on food poisoning: a time-series analysis of salmonellosis in ten European countries. Epidemiol Infect.

[16] D’Souza RM, Becker NG, Hall G, Moodie KB (2004). Does ambient temperature affect foodborne disease?. Epidemiol..

[17] McMichael AJ, Woodruff RE, Hales S (2006). Climate change and human health: present and future risks. Lancet..

[18] Czech Hydrometeorological Institute. Territorial air temperature, Historical data. http://portal.chmi.cz/historicka-data/pocasi/uzemni-teploty?l=en. Accessed 16 Jun 2019.

[19] Czech Statistical Office. 2019 https://www.czso.cz/csu/czso/obyvatelstvo_hu. Accessed 3 Jan 2020.

[20] Wood SN (2017). Generalized additive models: an introduction with R.

[21] R Core Team. R: A language and environment for statistical computing. R Foundation for Statistical Computing 2017. Vienna; 2017. https://www.R-project.org/. Accessed 7 Feb 2020.

[22] European Food Safety Authority, European Centre for Disease Prevention Control (2017). The European Union summary report on trends and sources of zoonoses, zoonotic agents and food-borne outbreaks in 2016. EFSA J.

[23] Chlebicz A, Śliżewska K (2018). Campylobacteriosis, salmonellosis, yersiniosis, and listeriosis as zoonotic foodborne diseases: a review. Int J Environ Res Public Health.

[24] Shah DH, Zhou X, Addwebi T, Davis MA, Orfe L, Call DR (2011). Cell invasion of poultry-associated Salmonella enterica serovar Enteritidis isolates is associated with pathogenicity, motility and proteins secreted by the type III secretion system. Microbiol..

[25] Akil L, Ahmad HA, Reddy RS (2014). Effects of climate change on Salmonella infections. Foodborne Pathog Dis.

[26] Zhang Y, Bi P, Hiller JE (2010). Climate variations and Salmonella infection in Australian subtropical and tropical regions. Sci Total Environ.

[27] Carlton EJ, Woster AP, DeWitt P, Goldstein RS, Levy K (2015). A systematic review and meta-analysis of ambient temperature and diarrhoeal diseases. Int J Epidemiol.

[28] Stephen DM, Barnett AG (2016). Effect of temperature and precipitation on salmonellosis cases in south-East Queensland, Australia: an observational study. BMJ Open.

[29] Yun J, Greiner M, Höller C, Messelhäusser U, Rampp A, Klein G (2016). Association between the ambient temperature and the occurrence of human Salmonella and campylobacter infections. Sci Rep.

[30] Milazzo A, Giles LC, Zhang Y, Koehler AP, Hiller JE, Bi P (2016). The effect of temperature on different Salmonella serotypes during warm seasons in a Mediterranean climate city, Adelaide, Australia. Epidemiol Infect.

